# The Association of Internet Use Intensity and Lifestyle Behaviors During the COVID-19 Pandemic: A Cross-Sectional Study in Chinese Adults

**DOI:** 10.3389/fpubh.2022.934306

**Published:** 2022-07-13

**Authors:** Yangyang Wang, Jian Xu, Tian Xie

**Affiliations:** ^1^China Institute for Urban Governance, Shanghai Jiao Tong University, Shanghai, China; ^2^School of International and Public Affairs, Shanghai Jiao Tong University, Shanghai, China; ^3^School of Media and Communication, Shanghai Jiao Tong University, Shanghai, China

**Keywords:** COVID-19, lifestyle behaviors, internet use intensity, public health in urban governance, public health in rural governance

## Abstract

The COVID-19 pandemic substantially increased the intensity of internet use in humans, which has made public opinion around health and public perceptions of it more vital, and this phenomenon has had a significant impact on human lifestyle behavior. This study used cross-sectional data during the COVID-19 pandemic to explore how internet use intensity influenced lifestyle behaviors among adults, and compared the differences between samples of different ages. The findings showed that the internet use intensity among adults increased the probability of physical activity, staying up late, and high-quality eating behaviors, and that they had a statistically significant positive association. Such associations were also found in independent younger, middle-aged, and older samples. However, the internet use intensity elevated the probability of body weight gain only in the independent samples of younger, middle-aged, and older adults. Besides, internet use intensity was able to increase the probability of smoking & drinking only among the younger sample. Notably, the effect of internet use intensity on lifestyle behaviors, including body weight gain, physical activity, staying up late, and a high-quality diet, was strongest among the elderly, followed by the middle-aged, and weakest among the younger. In the process of rural and urban governance regarding citizens' health, public health agencies should remind citizens to spend a reasonable amount of time on internet use to reduce the probability of unhealthy lifestyle behaviors and improve their physical health.

## Introduction

Since the outbreak of coronavirus in Wuhan of China at the end of 2019 (1), the WHO declared the COVID-19 pandemic on March 11, 2020, (2), followed by strict lockdown measures against COVID-19 in many countries worldwide (3), such as the United States (4), the United Kingdom (5), Germany (6), Italy (7), and Switzerland (8). Among them, China has implemented an extremely strict COVID-19 lockdown policy and travel restrictions (9–11). People were advised to stay at home and reduce contact to limit viral transmission. During this period, the frequency of Internet usage increased significantly. the internet can break through geographical limitations and realize instantaneous large-scale telematics exchanges without face-to-face contact, etc. These advantages have led to a massive migration of traditional information exchange (12), entertainment consumption (6), business offices (13), education and training (14, 15), online teaching (16), and other activity scenarios to interact in cyberspace, resulting in a significant increase in internet usage scenarios.

The increase in internet use intensity occurred in many countries such as Malaysia (17), Brazil (18), and Finland (19). During the COVID-19 outbreak in 2020, adults aged 60 and older in the United States used email and social media more than before and spent more time on their computers/tablets than ever before (20). Not only that, the intensity of internet use among Israeli adults increased after the COVID-19 pandemic, and the largest increase was in the use of chat software such as Zoom, Skype, and WhatsApp (21). During the COVID-19 pandemic in China, information such as health QR codes (22–24), trip QR codes, and vaccination information queries need to be accessed with the help of the internet, leading to a further increase in the demand for internet use. By December 2020, the number of internet users in mainland China has grown from 854 million in the first half of 2019 (25) to 989 million (26), a net increase of 135 million users, while the proportion of the internet user group aged 50 and above has also increased from 13.6% to 26.3% (25, 26). One study showed that time spent on the internet was much longer during the pandemic, and 72% of participants indicated an increasing dependence on the Internet (27). The per capita weekly internet hours have increased from 27.9 h (25) to 30.8 h in March 2020 during the strict lockdown period (28), the intensity of internet use among Chinese users is increasing significantly, with an average of more than 4 h of screen time per day (29).

The increase in internet use intensity also can promote users' access to more information related to health status and lifestyle (30, 31), for example, how to identify healthy foods (32), how to lose or control body weight (33, 34), increase fruit or vegetable intake (35) and optimize diet structure (36), increase willingness to physical activity (37, 38), and reduce alcohol intake (39). This information can drive lifestyle changes for information-exposed individuals, improve quality of life and lower depression scores (40). However, inappropriate internet use behaviors can also lead to unhealthy lifestyles, such as sedentary behavior, sleep deprivation, and cognitive impairment (31, 34). A study on Bangladeshi adolescents found that unhealthy internet use led to smoking and reduced physical activity (41). In addition, strict travel restrictions, home isolation, and lockdown measures during the COVID-19 pandemic are likely to lead to unhealthy lifestyle behaviors, including poorer quality of sleep loss (42–44), binge drinking, sedentary behavior, reduced physical activity, and increased alcohol and tobacco consumption (45). A study found that in the early stages of the COVID-19 pandemic there was approximately 25% deterioration in sleep quality (46), and a study in China found that the increase in the intensity of internet use was accompanied by a parallel increase in smoking and drinking behavior (47). In general, strict lockdown policies and the resulting increase in internet use have affected people's lifestyles and health behaviors (48, 49).

The worldwide COVID-19 pandemic has been ongoing for more than 2 years, which has profoundly affected the global economy, social development, and the functioning rhythms of countries. In the shock of the COVID-19 pandemic, individuals' internet use has become more frequent. The rising intensity of internet use has continued to shape people's daily life and health behaviors in terms of diet, physical activity, sleep, smoking, and drinking. However, existing studies have mostly focused on the mental health dimension (50–54), and children and adolescents groups (43, 55–58). There is a lack of research on how the increase in the intensity of internet use has affected the daily lifestyles of human adults, and whether the effect varies across age groups. On this basis, we conducted a study using nationwide level cross-sectional data from mainland China and examined the relationship between the internet use intensity and adults' lifestyle behaviors in the background of the COVID-19 pandemic.

## Methods

### Data and Participants

The data are from the wave 2020 of China Family Panel Studies (CFPS), funded by the 985 Program of Peking University and carried out by the Institute of Social Science Survey of Peking University, and are freely available at http://www.isss.pku.edu.cn/cfps/. CFPS database is a nationwide, large-scale, multidisciplinary social tracking survey covering 25 provinces/municipalities/autonomous regions in China Mainland, with a target sample size of 16,000 households and all household members in the sample. Wave 2020 of the CFPS database was conducted during the COVID-19 Pandemic and contained 28,590 samples, of which 25,193 were from adults aged 20 years or older. After filtering and cleaning the invalid sample, this study used 21,301 samples from the overall sample. There are 10,732 (50.38%) males and 10,569 (49.62%) females. 9,780 younger aged 20–44 years old, 6,649 middle-aged adults aged 45–59 years old, and 4,872 elderly adults aged 60–95 years old.

### Variables

#### Dependent Variable

In this study, the dependent variable included the lifestyle behaviors of body weight, physical exercise, staying up late, dietary nutrition, and smoking & drinking behaviors, which are coded as follows.

#### Body Weight

Using the height and weight data of individuals in the CFPS2020 database, the BMI value of the sample was calculated according to the BMI calculation formula [BMI = weight(kg)/height(m)^2^]. Then BMI was categorized as underweight (BMI < 18.5 kg/m^2^), normal weight (18.5 kg/ m^2^ ≤ BMI < 24 kg/m^2^), overweight (24 kg/m^2^ ≤ BMI < 28 kg/m^2^), and obesity (BMI ≥ 28 kg/m^2^) (59–62), and coded sequentially as “1” (underweight), “2” (normal weight), “3” (overweight), and “4” (obesity). The larger the value of the code, the more overweight the body is.

#### Physical Exercise

Use the question “How often did you participate in physical fitness and leisure activities in the past 12 months?” to measure physical exercise, which ranged from 1 (never participate) to 8 (twice a day or more). The larger the value of the code, the more frequent the physical exercise is.

#### Stay Up Late

Use the question “What time do you usually go to bed at night?” to measure stay up late. Then, code “sleep at 13~22” as “1,” code “sleep at 23~24” as “2,” code “sleep at 0~2 the next day” as “3”, code “sleep at 3~5 the next day”as “4,” and code “sleep at 6~12 the next day” as “5.” The greater the value of the code, the more serious the degree of staying up late.

#### Dietary Nutrition

Two questions were used to measure dietary nutrition, specifically, “In the past week, have you eaten meat?” and “In the past week, have you eaten fresh vegetables or fruits?.” Then, code “neither intake of meat, nor vegetables and fruits” as “1,” code “either intake of meat or vegetables and fruits “ as “2,” and code “intake of both meat, vegetables and fruits” as “3.” The larger the value of the code, the better the nutrition in the diet.

#### Smoking and Drinking

Two questions were used to measure smoking & drinking, specifically, “Have you smoked in the past month?” and “In the past 1 month, did you have alcohol more than 3 times a week? more than once a week?.” Then, code “neither smoking nor drinking” as “1,” code “ smoking or drinking” as “2,” and code “smoking and drinking” as “3.” The larger the value of the code, the more serious the unhealthy behavioral pattern.

### Independent Variable

#### Internet Use Intensity

Two questions were used to measure internet use intensity: “In general, how often do you use your mobile device to access the internet each day?,” and “In general, how often do you use your computer to access the internet each day?.” The usage time of computer internet access and mobile device internet access was summed by the hour and the calculated value was used to measure the intensity of internet use, a measurement method adopted by the previous studies (63–65). The more total time spent on the internet, the stronger the intensity of internet use.

### Control Variables

Based on the previous study (66–68), demographic characteristics variables, socioeconomic status variables, and health characteristics variable are selected as control variables. Specifically, demographic characteristics variables include gender (male, female), age (continuous variable), marriage status (1 = unmarried; 0 = married), education level (1 = not educated; 2 = primary school; 3 = junior high school; 4 = high school/senior high school college/technical school/vocational high school; 5 = college and above), and residence type (1 = city; 0 = rural). Socioeconomic status variables include annual income (continuous variable, and its unit is RMB 100,000), and health insurance (1 = have at least one; 0 = none). Health characteristics variable includes health conditions (1 = unhealthy; 2 = relatively unhealthy; 3 = normal; 4 = relatively healthy; 5 = very healthy).

### Statistical Analysis

Because each dependent variable in this study is a categorical variable and is coded for more than two types, it is suitable to choose the ordinal logistic regression model for regression analysis, and the specific model is as follows:


(1) $$ln\left(\frac{Probability_{j}}{1-Probability_{j}}\right)=-a_{j}+b_{0}Internet\;Use\;Intensity_{i}+\sum_{i=1}^{k}b_{i}x_{i}$$

In this designed model above, *j* indicates the number of categories of the dependent variable, Probability _*j*_ indicates the cumulative probability of the first j categories of the dependent variable, k is the number of control variables, and x is the vector of control variables. The estimated coefficient of the explanatory variable (β) indicates the probability of belonging to other categories compared to the category that it belongs to.

In addition, we used Stata17 to complete the descriptive statistical analysis, χ2 test, One-way ANOVA, bivariate correlations, and regression analysis. The multicollinearity test was first done and robust standard deviation was used for estimation to achieve the goal of eliminating statistical bias.

## Results

### Descriptive Statistics of All Variables

Table 1 shows the results of descriptive statistics for all variables, the mean value of internet use intensity was 2.415 (SD, 3.686) hours per day, and the value of 4.191 h per day for the younger adults, which was higher than the middle-aged sample (mean, 1.23 h) and the older sample (mean, 0.467 h). The statistical test showed that the statistical difference was significant (0.009, *p*< *0.001*), which indicates that the intensity of internet use was significantly stronger in the younger group than in the middle-aged and older groups. In terms of lifestyle behaviors, body weight (340.944, *p* < *0.001*), physical exercise (0.021, *p* < *0.001*), staying up late (0.024, *p* < *0.001*), dietary nutrition (524.085, *p* < *0.001*) and smoking & drinking (70.01, *p* < *0.001*) were significantly different among younger sample, middle-aged sample and older sample, which indicates that there are significant differences in lifestyle behaviors across different sample groups. In addition, all control variables were also significantly different in the younger adults, middle-aged sample, and the older sample and were statistically significant at 0.001.

**Table 1:** Descriptive statistics of all variables (*N* = 21,301).

**Variables**	**Full sample**	**Younger sample**	**Middle–aged sample**	**Older sample**	**χ2 test/F–test or t–test**
Body weight, mean (SD)	2.41 (0.75)	2.35 (0.76)	2.52 (0.71)	2.40 (0.75)	105.996^***^
Physical exercise, mean (SD)	2.59 (2.34)	2.48 (2.04)	2.49 (2.38)	2.97 (2.78)	80.924^***^
Stay up late, mean (SD)	1.35 (0.55)	1.54 (0.61)	1.25 (0.48)	1.12 (0.37)	1184.762^***^
Dietary nutrition, mean (SD)	2.85 (0.39)	2.91 (0.32)	2.82 (0.42)	2.77 (0.45)	229.303^***^
Smoking & drinking, mean (SD)	1.41 (0.63)	1.38 (0.61)	1.45 (0.66)	1.44 (0.65)	34.614^***^
Internet use intensity, mean (SD)	2.41 (3.69)	4.19 (4.42)	1.23 (2.16)	0.47 (1.31)	2707.865^***^
Gender, *N* (%)					10.777^***^
Female	10569 (49.62%)	4928 (50.39%)	3323 (49.98%)	2318 (47.58%)	
Male	10732 (50.38%)	4852 (49.61%)	3326 (50.02%)	2554 (52.42%)	
Age, mean (SD)	46.57 (15.67)	32.11 (6.70)	52.00 (4.07)	68.18 (5.83)	
Marriage status, *N* (%)					0.028^***^
Married	18707 (87.82%)	7339 (75.04%)	6553 (98.56%)	4815 (98.83%)	
Others	2594 (12.18%)	2441 (24.96%)	96 (1.44%)	57 (1.17%)	
Education level, *N* (%)					0.002^***^
Not educated	11844 (55.60%)	431 (4.41%)	6566 (98.75%)	4847 (99.49%)	
Primary school	1006 (4.72%)	976 (9.98%)	19 (0.29%)	11 (0.23%)	
Junior high school	3159 (14.83%)	3113 (31.83%)	39 (0.59%)	7 (0.14%)	
HJTV	3733 (17.52%)	3707 (37.90%)	19 (0.29%)	7 (0.14%)	
College and above	1559 (7.32%)	1553 (15.88%)	6 (0.09%)	0 (0.00%)	
Residence type, *N* (%)					146.645^***^
Rural	9842 (46.20%)	4082 (41.74%)	3357 (50.49%)	2403 (49.32%)	
Urban	11459 (53.80%)	5698 (58.26%)	3292 (49.51%)	2469 (50.68%)	
Annual income, mean (SD)	0.22 (0.39)	0.34 (0.47)	0.17 (0.30)	0.03 (0.12)	1233.886^***^
Health insurance, N (%)					111.898^***^
None	1997 (9.38%)	1132 (11.57%)	453 (6.81%)	412 (8.46%)	
Have at least one	19304 (90.62%)	8648 (88.43%)	6196 (93.19%)	4460 (91.54%)	
Health conditions, mean (SD)	3.07 (1.19)	3.39 (1.03)	2.91 (1.23)	2.63 (1.25)	808.390^***^
*N*	21,301	9,780	6,649	4,872	

*HJTV indicates High School/Junior High School/Technical School/Vocational High School. Younger adults are between the ages of 20 and 44, middle–aged adults are between the ages of 45 and 59, and older adults are between the ages of 60 and above*.

*** *p < 0.001*.

### Correlation Analysis Between Main Variables

Pearson correlation was used to conduct bivariate correlations for the main variables, and the results are shown in Table 2. Internet Use Intensity and Body Weight (*r* = −0.049, *p* < 0.001), Internet Use Intensity and Smoking & Drinking (*r* = −0.056, *p* < 0.001) were all significantly negatively correlated, but Internet Use Intensity and Physical Exercise (*r* = 0.114, *p* < 0.001), Internet Use Intensity and Stay Up Late (*r* = 0.340, *p* < 0.001), Internet Use Intensity and Dietary Nutrition (*r* = 0.153, *p* < 0.001) were all significantly positively correlated, which indicates that higher intensity of internet use is associated with lower rates of obesity and smoking & drinking, but higher rates of staying up late. Regarding lifestyle behavior variables, Stay Up Late was significantly negatively correlated with Body Weight (*r* = −0.033, *p* < 0.001), Smoking & Drinking was significantly negatively correlated with Physical Exercise (*r* = −0.037, *p* < 0.001), Dietary Nutrition was not statistically correlated with Body Weight, and there were statistically positive correlations between all other variables in lifestyle behaviors.

**Table 2:** Correlation analysis between main variables (*N* = 21,301).

**Variables**	**Internet use intensity**	**Body weight**	**Physical exercise**	**Stay up late**	**Dietary nutrition**	**Smoking & drinking**
Internet use intensity	–					
Body weight	−0.049^***^	–				
Physical exercise	0.114^***^	0.037^***^	–			
Stay up late	0.340^***^	−0.033^***^	0.063^***^	–		
Dietary nutrition	0.153^***^	0.009	0.100^***^	0.120^***^	–	
Smoking & drinking	−0.056^***^	0.043^***^	−0.037^***^	0.035^***^	0.049^***^	–

*** *p < 0.01*.

### Internet Use Intensity and Body Weight

The large increase in the intensity of internet use brings about a decrease in physical activity time. Internet–addicted users tend to spend long hours surfing the internet, playing games, browsing short videos and social media, and so on, which is likely to lead to the occurrence of overweight or obesity. Table 3 shows the results of the analysis using the ordinal logistic regression model, we constructed four regression models with a full sample, younger sample, middle–aged sample, and older sample, and analyzed the effect of internet use intensity on body weight. The size of the total sample is 21,301, the size of the younger sample is 9,780, the middle–aged sample is 6,649, and the older sample is 4,872.

**Table 3:** Results of the relationship between internet use intensity and body weight (*N* = 21,301).

**Variables**	**Full sample**	**Younger sample**	**Middle–aged sample**	**Older sample**
	**Model 1**	**Model 2**	**Model 3**	**Model 4**
Internet use intensity	0.006	0.011^**^	0.023^**^	0.062^**^
	(1.171)	(2.154)	(1.997)	(2.524)
Gender (ref: female)	0.423^***^	0.918^***^	0.148^***^	−0.141^**^
	(15.343)	(21.721)	(2.996)	(−2.482)
Age	−0.007^***^	0.040^***^	−0.004	−0.032^***^
	(−4.603)	(9.983)	(−0.645)	(−6.538)
Marriage status (ref: married)	−1.036^***^	−0.776^***^	−0.230	−0.414
	(−19.501)	(−12.313)	(−0.966)	(−1.485)
Education level	−0.125^***^	−0.021	−0.164^**^	−0.130
	(−7.862)	(−0.887)	(−2.060)	(−0.908)
Residence type (ref: rural)	0.209^***^	0.060	0.127^***^	0.464^***^
	(7.568)	(1.408)	(2.595)	(7.990)
Annual income	0.112^***^	−0.071	0.049	0.091
	(3.040)	(−1.611)	(0.575)	(0.394)
Health insurance (ref: none)	0.061	0.019	0.163^*^	−0.082
	(1.294)	(0.285)	(1.733)	(−0.847)
Health conditions	−0.044^***^	−0.109^***^	−0.013	0.037
	(−3.591)	(−5.078)	(−0.672)	(1.615)
Observations	21,301	9,780	6,649	4,872
Wald chi2	922.41	1098.29	34.08	150.1
Pseudo R^2^	0.021	0.052	0.003	0.014
Log pseudolikelihood	−22776.192	−10186.886	−69914.552	−5315.633

*Robust standard errors in parentheses;*

*** *p < 0.01,*

** *p < 0.05*.

In this part of the analysis, we analyzed the effect of internet use intensity and control variables on body weight with the help of ordinal logistic regression model regression. Before constructing the model, we conducted a multicollinearity test for all variables, including the overall sample, the younger sample, the middle–aged sample, and the older sample. The results showed that the VIF values of all samples did not exceed 3, indicating that there was no serious multicollinearity problem. The regression results showed that the effect of internet use intensity on body weight was not statistically significant. However, the results of the subsample regression tests were statistically significant at the 5% level, indicating that internet use intensity significantly affected body weight in different age groups, which were the young adult sample (β = 0.011, *p* < 0.05), the middle–aged sample (β = 0.023, *p* < 0.05) and the elderly sample (β = 0.062, *p* < 0.05). The comparison revealed that the stronger the intensity of internet use, the greater the probability of body weight gain, this finding has been found in the different age groups. And the probability of body weight gain in the elderly far exceeded that of the middle–aged and younger sample.

It's widely believed that screen time is responsible for obesity. A system review designed a meta–analysis, showing that internet use was positively related to increased odds of being overweight and obese (69). Besides, Internet addiction has also been regarded as a predictor of body weight gain (70). Hypokinesis is the nature of internet (71), high–intensity internet use leads to sedentary, reduced energy expenditure, and lack of physical exercise, which may finally result in body weight gain (71–73). One study has a different finding that there was no significant association between internet use and obesity among female adolescents in Switzerland (64).

### Internet Use Intensity and Physical Exercise

A significant increase in internet use intensity not only allows the exposure to a lot of health information, but also is likely to be attracted by useful information on physical fitness and exercise, but may also lead to Internet addiction due to indulgence in online games, making the willingness to physical exercise lower. Table 4 shows the results of the analysis using the ordinal logistic regression model, we built four regression models separately to analyze the effect of internet use intensity on body weight, including the results of the overall sample and sub–sample analysis. Before the empirical analysis, a multicollinearity test was conducted and the results showed that the VIF values of all models did not exceed 3.

**Table 4:** Results of the relationship between internet use intensity and physical exercise (*N* = 21,301).

**Variables**	**Full sample**	**Younger sample**	**Middle–aged sample**	**Older sample**
	**Model 5**	**Model 6**	**Model 7**	**Model 8**
Internet use intensity	0.048^***^	0.018^***^	0.135^***^	0.174^***^
	(12.612)	(3.862)	(10.259)	(8.240)
Gender (ref: female)	0.079^***^	0.246^***^	−0.252^***^	0.238^***^
	(2.741)	(5.917)	(−4.528)	(3.847)
Age	0.030^***^	0.007^*^	0.040^***^	0.001
	(18.787)	(1.791)	(6.088)	(0.185)
Marriage status (ref: married)	0.737^***^	0.689^***^	0.091	0.011
	(18.263)	(12.322)	(0.399)	(0.038)
Education level	0.183^***^	0.570^***^	0.153	0.001
	(11.844)	(21.874)	(1.647)	(0.010)
Residence type (ref: rural)	0.785^***^	0.334^***^	0.865^***^	1.033^***^
	(25.014)	(7.321)	(14.881)	(16.056)
Annual income	0.131^***^	0.079^**^	0.410^***^	−0.350
	(4.624)	(2.261)	(5.295)	(−1.129)
Health insurance (ref: none)	0.309^***^	0.237^***^	0.349^***^	0.307^***^
	(6.227)	(3.571)	(3.008)	(2.612)
Health conditions	0.037^***^	0.034	−0.003	0.069^***^
	(2.819)	(1.599)	(−0.108)	(2.758)
Observations	21,301	9,780	6,649	4,872
Wald chi2	2039.46	1350.8	542.45	441.54
Pseudo R^2^	0.035	0.057	0.043	0.043
Log pseudolikelihood	−25955.227	−12488.561	−6983.610	−5071.297

*Robust standard errors in parentheses;*

*** *p < 0.01,*

** *p < 0.05,*

* *p < 0.1*.

Table 4 presents the results of the regression analysis. The overall sample test in model 5 indicated that internet use intensity had a positive effect on physical exercise (β = 0.049, *p* < 0.01), which was significant at the 1% level of significance. The results of the regression tests for the subsamples were statistically significant at the 1% level. This indicates that internet use intensity significantly affects physical exercise in different age groups, namely the young adult sample (β = 0.018, *p* < 0.01), the middle–aged sample (β = 0.135, *p* < 0.01), and the older sample (β = 0.174, *p* < 0.01). The comparison reveals that the strength of the positive effect of internet use intensity on physical exercise in the older sample and the middle–aged sample is significantly greater than this effect in the younger sample. This finding is different from previous research that those who have more screen time were proved to have reduced physical activity (74), and overuse of internet such as internet addiction has a significant negative effect on physical activity (75). A possible reason may be that China's “Internet + Exercise” public health policy calls for people to actively participate in physical exercise through the Internet (76). Physical activity was performed via the Internet during covid−19 due to a decrease in outdoor activity.

### Internet Use Intensity and Stay Up Late

Before conducting the empirical analysis, we excluded the effect of multicollinearity because the VIF values of all models did not exceed 3. Table 5 reports the ordinal logistic regression results of internet use intensity affecting staying up late. In the full sample, internet use intensity positively affects staying up late (β = 0.083, *p* < 0.01), which is significant at the 1% level of significance. This indicates that the higher the intensity of internet use, the later the time to go to bed each night. The regression results for the subsamples were also consistent, with the younger sample (β = 0.061, *p* < 0.01), the middle–aged sample (β = 0.164, *p* < 0.01), and the older sample (β = 0.252, *p* < 0.01) all significant at the 1% level of significance. However, the comparison revealed that the internet use intensity positively affected staying up late more strongly in the older sample and the middle–aged sample than in the younger sample.

**Table 5:** Results of the relationship between internet use intensity and stay up late (*N* = 21,301).

**Variables**	**Full sample**	**Younger sample**	**Middle–aged sample**	**Older sample**
	**Model 9**	**Model 10**	**Model 11**	**Model 12**
Internet use intensity	0.083^***^	0.061^***^	0.164^***^	0.252^***^
	(16.131)	(10.894)	(11.563)	(8.335)
Gender (ref: female)	0.311^***^	0.407^***^	0.193^***^	0.166
	(9.289)	(9.265)	(3.031)	(1.737)
Age	−0.034^***^	−0.028^***^	−0.022^***^	−0.050^***^
	(−18.482)	(−6.761)	(−2.987)	(−5.601)
Marriage status (ref: married)	0.611^***^	0.705^***^	0.281	−0.471
	(12.082)	(11.374)	(1.110)	(−0.754)
Education level	0.074^***^	0.220^***^	0.192^**^	−0.459
	(3.995)	(8.480)	(2.069)	(−1.474)
Residence type (ref: rural)	0.648^***^	0.450^***^	0.820^***^	0.693^***^
	(18.897)	(9.824)	(12.656)	(6.773)
Annual income	0.329^***^	0.257^***^	0.347^***^	−0.667
	(8.187)	(5.675)	(3.468)	(−1.396)
Health insurance (ref: none)	−0.029	−0.075	−0.106	0.164
	(−0.523)	(−1.112)	(−0.890)	(0.874)
Health conditions	−0.098^***^	−0.123^***^	−0.086^***^	−0.046
	(−6.626)	(−5.731)	(−3.422)	(−1.248)
Observations	21,301	9,780	6,649	4,872
Wald chi2	3977.31	1434.13	501	203.39
Pseudo R^2^	0.15	0.095	0.071	0.059
Log pseudolikelihood	−13249.784	−7648.836	−3719.038	−1759.094

*Robust standard errors in parentheses;*

*** *p < 0.01,*

** *p < 0.05*.

Previous studies have found that the longer the duration of internet use (>5 h) among adolescents, the higher the likelihood of sleep problems such as insomnia, shorter sleep duration, late sleep onset, and poor sleep quality (55). Addictive social media use is a potential risk factor for insomnia in adolescents (56), which is significantly and positively associated with insomnia (77) and is more likely to occur in females than in males (78). Longitudinal studies found frequent social media use to be a significant influence on adolescents' poor sleep (43), and screen media contributed to late nights and reduced sleep duration (79, 80). The longer the time spent in bed using social media, the more likely insomnia, anxiety, and short sleep duration were experienced on weekday nights (81). Our study further confirms that the longer the adult group's Internet use, the higher the probability of staying up late. In addition, in terms of control variables, the type of residence and physical health condition also had a significant effect on staying up late, with samples living in cities being more likely to stay up late and samples with poor physical health conditions being more likely to stay up late.

### Internet Use Intensity and Dietary Nutrition

After excluding the effect of multicollinearity, we conducted an empirical analysis. Table 6 presents the ordinal logistic regression results for the impact of internet use intensity on diet nutrition. The regression results of the ordinal logistic model showed that internet use intensity had a positive effect (β = 0.130, *p* < 0.01), which was significant at the 1% significance level. In the regression results of the sub–samples, all were statistically significant at the 1% level, indicating that internet use intensity had a significant and positive effect on dietary nutrition in different age groups, i.e., younger adults (β = 0.095, *p* < 0.01), middle–aged sample (β = 0.177, *p* < 0.01) and older sample (β = 0.290, *p* < 0.01). The comparison revealed that the internet use intensity positively affected dietary nutrition to the degree that it was significantly stronger in the older sample and the middle–aged sample than in the younger sample.

**Table 6:** Results of the relationship between internet use intensity and dietary nutrition (*N* = 21,301).

**Variables**	**Full sample**	**Younger sample**	**Middle–aged sample**	**Older sample**
	**Model 13**	**Model 14**	**Model 15**	**Model 16**
Internet use intensity	0.130^***^	0.095^***^	0.177^***^	0.290^***^
	(8.822)	(5.624)	(5.745)	(5.022)
Gender (ref: female)	0.305^***^	0.500^***^	0.164^**^	0.280^***^
	(7.255)	(6.266)	(2.382)	(3.844)
Age	0.001	0.006	0.005	0.003
	(0.489)	(0.773)	(0.601)	(0.549)
Marriage status (ref: married)	−0.312^***^	−0.215	−0.418	−0.423
	(−3.450)	(−1.798)	(−1.653)	(−1.365)
Education level	0.248^***^	0.382^***^	0.159	−0.116
	(8.580)	(8.958)	(1.037)	(−0.430)
Residence type (ref: rural)	0.471^***^	0.325^***^	0.401^***^	0.570^***^
	(11.039)	(4.077)	(5.696)	(7.586)
Annual income	1.077^***^	0.684^***^	1.405^***^	1.937^***^
	(8.220)	(3.991)	(6.357)	(3.337)
Health insurance (ref: none)	0.307^***^	0.453^***^	0.178	0.186
	(4.599)	(4.389)	(1.411)	(1.530)
Health conditions	0.040^**^	−0.070	0.073^***^	0.067^**^
	(2.260)	(−1.862)	(2.592)	(2.261)
Observations	21,301	9,780	6,649	4,872
Wald chi2	881.92	38.78	203.16	147.59
Pseudo R^2^	0.075	0.078	0.044	0.036
Log pseudolikelihood	−8809.987	−2889.197	−3198.019	−2675.992

*Robust standard errors in parentheses;*

*** *p < 0.01,*

** *p < 0.05*.

Our findings regarding the adult groups are consistent with similar studies. A similar study found that the longer the duration of internet use among Egyptian children and adolescents during COVID−19, the more likely they were to reduce their intake of vegetables, fruits, or protein (82). The Instagram Social Network can influence adolescents' eating behaviors (83) and shape eating trends (84). The duration of Instagram is significantly associated with dietary behavior (85). Food marketing advertisements in social media also influence the dietary choices of children and adolescents (86). In addition, in terms of control variables, type of residence, annual income, and physical health condition had a significant effect on dietary nutrition, with samples living in urban areas, higher annual income, and better physical health conditions having a greater probability of increasing nutrition in the diet.

### Internet Use Intensity and Smoking and Drinking

Our regression analysis was performed after excluding the effect of multicollinearity. Table 7 reports the results of the regression analysis of internet use intensity of smoking & drinking. We analyzed the effect of internet use intensity and control variables on smoking & drinking with the help of an ordinal logistic regression model. The results showed that the effect of internet use intensity on smoking & drinking was not statistically significant in the overall sample. However, in the results of the subsample regression, internet use intensity positively affected smoking & drinking only in the younger sample (β = 0.015, *p* < 0.05) and was statistically significant at the 5% level. This finding indicates that the internet use intensity can only increase the probability of smoking or drinking in the younger group. One possible explanation may be that, as people age, middle–aged and elderly adults may receive more health information and warnings about the risks associated with smoking and drinking, making them generally stay away from smoking or drinking (87).

**Table 7:** Results of the relationship between internet use intensity and smoking & drinking (*N* = 21,301).

**Variables**	**Full sample**	**Younger sample**	**Middle–aged sample**	**Older sample**
	**Model 17**	**Model 18**	**Model 19**	**Model 20**
Internet use intensity	−0.004	0.015^**^	−0.018	−0.052
	(−0.653)	(2.187)	(−1.237)	(−1.643)
Gender (ref: female)	3.624^***^	4.021^***^	3.799^***^	2.938^***^
	(68.804)	(42.501)	(41.059)	(32.933)
Age	−0.009^***^	0.019^***^	0.017^**^	−0.019^***^
	(−4.822)	(3.732)	(2.217)	(−2.991)
Marriage status (ref: married)	−0.489^***^	−0.347^***^	−0.173	−0.171
	(−8.173)	(−4.682)	(−0.692)	(−0.543)
Education level	−0.242^***^	−0.427^***^	−0.009	0.137
	(−11.500)	(−13.520)	(−0.094)	(0.482)
Residence type (ref: rural)	−0.076^**^	0.109	−0.126	−0.174^**^
	(−2.112)	(1.922)	(−1.958)	(−2.450)
Annual income	0.178^***^	0.132^**^	0.131	0.918^***^
	(3.564)	(2.178)	(1.309)	(3.417)
Health insurance (ref: none)	−0.195^***^	−0.166^**^	−0.144	−0.284^**^
	(−3.250)	(−1.997)	(−1.091)	(−2.451)
Health conditions	0.092^***^	0.045	0.104^***^	0.143^***^
	(6.039)	(1.741)	(4.133)	(5.110)
Observations	21,301	9,780	6,649	4,872
Wald chi2	5112.60	1928.86	1763.29	1171.70
Pseudo R^2^	0.274	0.302	0.295	0.216
Log pseudolikelihood	−12685.891	−5321.270	−4029.871	−3222.972

*Robust standard errors in parentheses;*

*** *p < 0.01,*

** *p < 0.05*.

Similar studies found that: more frequent social media use was associated with a greater willingness to accept e–cigarettes (57), and problematic social networking site usage increased the probability of smoking among adolescents (58). A study on Norwegian adolescents found that more time spent on social media increased episodic heavy drinking behavior (88), and a Canadian study also found that longer social media use was associated with a higher likelihood of alcohol use among adolescents (89). As many as 19 studies have confirmed that higher alcohol–related social media engagement is associated with higher self–reported drinking and alcohol–related problems (90). Moreover, the more time spent on social media use behaviors such as online social networking (91), Frequent electronic media communication with friends (92), the greater the likelihood of adolescent episodic alcohol use (93, 94). Consistent with these studies, our study found that the longer the younger's internet use time, the higher the probability of smoking & drinking. However, there was no significant association in middle–aged and elderly samples. In addition, type of residence, annual income, health insurance, and physical health condition had significant effects on smoking & drinking. It's worth noting that tobacco and alcohol consumption tended to decrease among those who lived in cities and had health insurance. People with higher annual income and better physical health conditions tend to increase their intake of cigarettes and alcohol.

### Robustness Test

The purpose of the robustness test is to examine the stability of the empirical test results. That is, whether the effect of the independent variable on the dependent variable remains robust when the measurement of the variable is changed, or when another regression model is chosen. If the measurement structure of the independent or dependent variable is changed, another empirical test is conducted, and the positive and negative directions and significance levels of the variables are found to have changed, indicating that the results are not robust. In short, the reliability of the conclusions can be strengthened by this process. However, there is no standardized stability test specification, and subsample regression, reselecting the regression model, performing variable replacement, and changing the sample size are all commonly used in robustness tests. Considering the limitations of the data, we chose an OLS model to re–test the effect of internet use intensity on lifestyle behaviors.

Table 8 shows the results of the robustness tests using the OLS model. Among all the OLS models, R–squared and F statistics meet the statistical requirements and the model fitness is good. The results show that Table 3 (Internet Use Intensity and Body Weight), Table 4 (Internet Use Intensity and Physical Exercise), Table 5 (Internet Use Intensity and Stay Up Late), and Table 6 (Internet Use Intensity and Dietary Nutrition) are consistent with the results of the robustness test, which proves the reliability of the findings. However, the regression results of Table 7 (Internet Use Intensity and Smoking & Drinking) differed from the results of the robustness test. Specifically, the results of the robustness test showed that the internet use intensity of the younger sample not significantly affected smoking & drinking behavior, indicating the sensitivity of smoking & drinking behavior of the older sample. Overall, the regression test using the ordinal logistic model is robust and supports the reliability of the findings.

**Table 8:** Results of robustness tests.

**Variables**	**Full sample**	**Younger adults**	**Middle–aged adults**	**Older adults**
Body weight (dependent variable)				
Internet use intensity	0.003	0.004^**^	0.010^**^	0.024^**^
	(1.450)	(2.340)	(2.251)	(2.354)
Physical exercise (dependent variable)				
Internet use intensity	0.061^***^	0.019^***^	0.178^***^	0.290^***^
	(11.692)	(3.641)	(10.378)	(7.916)
Stay up late (dependent variable)				
Internet use intensity	0.023^***^	0.017^***^	0.042^***^	0.045^***^
	(15.900)	(10.673)	(11.170)	(6.925)
Dietary nutrition (dependent variable)				
Internet use intensity	0.007^***^	0.005^***^	0.014^***^	0.026^***^
	(11.308)	(6.814)	(7.647)	(6.847)
Smoking & drinking (dependent variable)				
Internet use intensity	−0.001	0.002	−0.004	−0.014
	(−0.477)	(1.804)	(−1.145)	(−1.943)
Observations	21,301	9,780	6,649	4,872

*Robust standard errors in parentheses;*

*** *p < 0.01,*

** *p < 0.05.*

*The control variables are gender (ref: female), age, marriage status (ref: married), education level, residence type (ref: rural), annual income, health insurance (ref: none), and health conditions*.

## Discussion

The COVID−19 pandemic led to the imposition of strict lockdown measures in many countries (3–8), of which China imposed a much longer period (9–11). The impact of blocking measures has led to a significant increase in internet use intensity in China (95). A 2.9 h increase per person per week during the peak period (25, 28) has influenced lifestyle behaviors. This study uses data from the 2020 wave of CFPS to empirically analyze a sample of 21,301 individuals in mainland China.

Across the full sample, internet use intensity positively influenced physical exercise, staying up late, and diet nutrition in lifestyle. This finding suggests that the stronger internet use intensity, the greater the likelihood of body weight gain, the more frequency of physical exercise, the higher probability of staying up late, and the greater emphasis on increasing vegetables and fruits or meat in the diet. A study from Libya found that people who used the internet multiple times a day during the COVID−19 pandemic fell asleep later and were more likely to have sleep disturbances than those who used the internet infrequently (96). In a similar case in Mexico, increased internet use among adults was accompanied by later sleep (97), which is consistent with our findings. However, the Brazilian study showed that the increased intensity of internet use was accompanied by a decrease in physical activity, and vegetables and fruits in the diet (18), which contradicts our findings, but the Brazilian study did not perform a statistical analysis of these variables.

Furthermore, internet use intensity had no significant effect on body weight and smoking & drinking. This indicates that internet use intensity is not associated with body weight gain, this finding is supported by a similar study (98). However, a study with a sample of children contradicts this finding (99). In this case, the effect of internet use intensity on body weight was not significant, but the age–graded sample was significant, which is probably due to the data division structure.

A further comparison of samples from three groups of young, middle–aged, and older adults revealed that internet use intensity positively influenced staying up late, dietary nutrition, and physical exercise in lifestyle. Increased intensity of internet use among young Malaysians was accompanied by an increase in the incidence of staying up late (100). During the COVID−19 pandemic, the greater the intensity of internet use, the more negative emotional experiences were felt through the internet, and the greater the likelihood of emotional eating (101). Contrary to our findings, a study in Brazil showed that the increased intensity of internet use was accompanied by a decrease in physical activity in different age groups (18), possibly because the Brazilian data were investigated in only one city and were conducted at the beginning of the COVID−19 pandemic, a time when lockdown measures blocked physical activity from performing. Based on a national sample, the current study found that internet use intensity was positively associated with physical exercise in different age groups, a possible explanation may be that the internet provides a platform for individuals to obtain exercise information, transfer exercise data, and share exercise results during the pandemic.

Internet use intensity among younger adults positively influenced smoking & drinking as a part of lifestyle, but this association was not found among middle–aged and older adults. A study of young adults in Bangladesh showed that problematic internet use was positively associated with smoking behavior (41, 102). As for drinking consumption, a study of Brazilian adults found that increased time spent on the internet was accompanied by increased smoking and drinking behavior (18). A Finnish study pointed out that greater participation in social media was associated with a higher risk of drinking (19). And a study of older Americans found that elderly adults' alcohol intake did not increase during the COVID−19 pandemic (20). These studies support our findings but differ from the phenomenon reflected in one study (47), Although previous studies have been consistent with our findings, these studies have not been subjected to explicit statistical testing.

It is particularly notable that the effects of internet use intensity on lifestyle behaviors, including body weight gain, physical activity, staying up late, and a high–quality diet. Internet use intensity positively affects physical activity and weight gain, while physical activity generally reduces weight. One possible explanation is that internet use fails to improve exercise behaviors to the extent that it can affect body weight (103). Besides, these associations between internet use intensity and lifestyle behaviors were strongest in the elderly sample, followed by the middle–aged sample, and then in the younger sample. A likely explanation is that the time of internet access, as the elderly increased their internet access substantially after the COVID−19 pandemic (18, 20, 25, 26), making the shock effect of the internet much larger than that of the middle–aged and younger sample, while the younger accessed the internet early enough that this shock effect has been attenuated. Compared with other age groups, the primary purpose for the elderly to use the internet is for health reasons (104). And the health information which Chinese elderly obtained from the internet was mainly related to diet (63.1%) and exercise (47.1%) (105). Therefore, older adults who use the internet more intensively are more likely to have physical activity and a high–quality diet. A survey showed that 42% of the elderly take more than half an hour to fall asleep, which is significantly higher than that of young people (106). The elderly with a high frequency of Internet use may have a longer bedtime internet use, which increases the time in staying up late. Besides, Higher levels of internet use may result in sedentary and decreased outside activities among older adults (107), which may affect body weight gain. Generally, moderate and efficient Internet use is beneficial for older adults.

The findings of this study also responded to Link and Phelan's fundamental–causes theory (108). Link and Phelan argue that social condition, rather than medical, natural, or biological conditions, is the underlying cause of health differentiation in humans. While medical technology continues to evolve, the social condition has a continuous impact on people's quality of health (108). The COVID−19 pandemic has accelerated human internet access worldwide and increased the intensity of human Internet use. This in itself has profoundly influenced social conditions, making internet use an important social factor. The increased intensity of internet use is often accompanied by obesity and staying up late. But at the same time, physical activity and the quality of diet have also increased. These lifestyle behaviors are closely related to healthcare and further enrich the fundamental–causes theory.

## Limitations

In the research design and study process, we ensured the reliability of our findings in three ways. Firstly, we selected data from the wave 2020 of CFPS, a large survey based in mainland China that has lasted for seven issues since 2010. The quality of the data was ensured by the guidance and assistance from the Center for Social Research at the University of Michigan in terms of survey design and technical support (109, 110). Secondly, during the empirical testing process, we ensured the reliability and robustness of the empirical results by selecting control variables, regressing subsamples by age groups, and using OLS models for robustness testing. Finally, our data were collected in 2020, and the latest data application was opened in December 2021, and the data can reflect the latest situations. Moreover, the size of the sample size is 21,301, and its representative is very strong. This is a significant strength of this study.

However, there remain limitations to this study. Firstly, as the data in this study are cross–sectional, our empirical analysis can only reflect correlations but not cause and effect relationships, and some models have a low R^2^ and limited explanatory power. Secondly, due to the limitation of the database, some variables were not measured most perfectly during the measurement of dependent and independent variables, for example, the data on sleep duration were too many missing, and the time of falling asleep had to be chosen instead. Thirdly, this study only examined the grouping from the perspective of age, and further analysis is still needed from the perspective of urban–rural characteristics, gender characteristics, socioeconomic status characteristics, and so on. Fourthly, although our research shows that the intensity of internet use affects lifestyle behaviors, however, we ignored the impact of inappropriate internet use on lifestyle behaviors. It's acknowledged that internet overuse and problematic internet use are associated with lifestyle behaviors such as sedentary behavior (111), lack of exercise (75), sleep disturbance (112), eating disorder (113). It's necessary to explore further studies to examine the relationship between inappropriate internet use and unhealthy life behaviors. Last but not least, we should consider the influence of more control variables like family technology devices, and confounding variables such as anxiety, stress, and other psychological factors (27).

## Conclusions

Over the period of the COVID−19 pandemic, internet use intensity significantly influenced lifestyle behaviors among adults. Our research confirmed the previous findings that the enhanced internet use intensity was accompanied by an increased probability of staying up late and obesity in different age groups. Besides, this study had some different findings. Contrary to previous studies, the present study found that higher levels of internet use were positively associated with physical activity and high–quality dietary behaviors in Chinese adults. Additionally, only the elevated intensity of internet use among the younger increased the probability of smoking & drinking. It is particularly important to note that the effect of internet use intensity on lifestyle behavior is the strongest in the elderly sample, followed by the middle–aged sample, and weakest in the young adult sample. These findings suggest that attention should be paid to the impact of internet use intensity on negative life behaviors such as staying up late, smoking, and drinking. At the same time, appropriate use of the internet should be encouraged to promote physical exercise and maintain a healthy diet especially in older adults.

The findings of the study give us new insights. While the internet use intensity is universally increasing, public health agencies should remind the citizens to invest moderate time in internet use to avoid the occurrence of internet addictive behaviors during the process of rural and urban governance. Furthermore, the increased internet use intensity is often accompanied by access to health information, making adults pay more attention to physical activity and proper diet. In the future, internet use intensity will become an important factor affecting adults' health and is likely to lead to the occurrence of health disparities and health inequalities among adults.

## Data Availability Statement

The original contributions presented in the study are included in the article/supplementary material, further inquiries can be directed to the corresponding author.

## Author Contributions

YW: framework, model analyses, data curation, and writing—original draft. TX: writing—review and editing and data curation. JX: supervision and funding acquisition. All authors have read and agreed to the published version of the manuscript.

## Conflict of Interest

The authors declare that the research was conducted in the absence of any commercial or financial relationships that could be construed as a potential conflict of interest.

## Publisher's Note

All claims expressed in this article are solely those of the authors and do not necessarily represent those of their affiliated organizations, or those of the publisher, the editors and the reviewers. Any product that may be evaluated in this article, or claim that may be made by its manufacturer, is not guaranteed or endorsed by the publisher.
